# Comparative Transcriptomic Analysis Reveals Diverse Expression Pattern Underlying Fatty Acid Composition among Different Beef Cuts

**DOI:** 10.3390/foods11010117

**Published:** 2022-01-04

**Authors:** Tianliu Zhang, Qunhao Niu, Tianzhen Wang, Xu Zheng, Haipeng Li, Xue Gao, Yan Chen, Huijiang Gao, Lupei Zhang, George E. Liu, Junya Li, Lingyang Xu

**Affiliations:** 1 Laboratory of Molecular Biology and Bovine Breeding, Institute of Animal Science, Chinese Academy of Agricultural Sciences, Beijing 100193, China; zhangtianliu92@foxmail.com (T.Z.); nqh_5195@163.com (Q.N.); tianzhenwang5@163.com (T.W.); zhengxu0131@163.com (X.Z.); Suixin6516@163.com (H.L.); gaoxue76@126.com (X.G.); chenyan0204@163.com (Y.C.); gaohuijiang@caas.com (H.G.); zhanglupei@caas.cn (L.Z.); lijunya@caas.cn (J.L.); 2 Animal Genomics and Improvement Laboratory, United States Department of Agriculture-Agricultural Research Services, Beltsville, MD 20705, USA; George.liu@usda.gov

**Keywords:** fatty acids, transcriptome, differentially expressed genes, co-expression gene network, beef cuts

## Abstract

Beef is an important dietary source of quality animal proteins and amino acids in human nutrition. The fatty acid composition is one of the indispensable indicators affecting nutritional value of beef. However, a comprehensive understanding of the expression changes underlying fatty acid composition in representative beef cuts is needed in cattle. This study aimed to characterize the dynamics of fatty acid composition using comparative transcriptomic analysis in five different type of beef cuts. We identified 7545 differentially expressed genes (DEGs) among 10 pair-wise comparisons. Co-expression gene network analysis identified two modules, which were significantly correlated with 2 and 20 fatty acid composition, respectively. We also identified 38 candidate genes, and functional enrichment showed that these genes were involved in fatty acid biosynthetic process and degradation, PPAR, and AMPK signaling pathway. Moreover, we observed a cluster of DEGs (e.g., *SCD*, *LPL*, *FABP3*, and *PPARD*) which were involved in the regulation of lipid metabolism and adipocyte differentiation. Our results provide some valuable insights into understanding the transcriptome regulation of candidate genes on fatty acid composition of beef cuts, and our findings may facilitate the designs of genetic selection program for beneficial fatty acid composition in beef cattle.

## 1. Introduction

Beef is an important animal origin food source which can provide essential nutrients including essential amino acids, unsaturated fatty acids, minerals, and various vitamins for human health [1]. The nutritional value of meat has received tremendous attention in recent years [2]. Fatty acid composition is one of the important indicators affecting beef quality including flavor, juiciness, tenderness, and taste, and it strongly influences consumer’s preferences [3]. Many studies revealed that diets rich in monounsaturated fatty acids (MUFA) and polyunsaturated fatty acids (PUFA) have important effects on human health, such as decreasing the risk of cardiovascular disease, diabetes, and premature death [4].

The accumulation of fatty acid composition is regulated by various biological processes such as synthesis, transport, degradation, and beta-oxidation processes [5]. Several previous studies have been carried out to identify candidate variants and genes (e.g., *SCD*, *CD36*, *ACADM*, *PPARGC1A*, *LPL*), which are associated with fatty acid composition in cattle [6,7]. In addition, many studies explored the genetic basis of the fatty acids in muscles using multi-omics approaches including transcriptomic, proteomic, and metabolomic analyses. For instance, transcriptome analysis of bovine longissimus dorsi and skeletal muscle revealed that the differentially expressed genes (DEGs) were involved in various pathways (e.g., fatty acid degradation, fatty acid beta-oxidation, PPAR and AMPK signal pathways), which are related to fatty acid composition [8]. Metabolome analyses of longissimus dorsi and semitendinosus have been carried out to assess relations among breeds, and significant breed difference was observed in fatty acid and meat quality profiles [9]. Moreover, the distribution of fatty acids and phospholipid varied in different beef cuts, and beef cuts with high phospholipids content had high levels of ω-3 fatty acids and low levels of SFA [10]. Therefore, a comprehensive transcriptomic analysis of candidate genes for different beef cuts using high-throughput RNA sequencing can help to elucidate the potential gene expression regulation related to fatty acid composition.

To explore the transcription regulation changes for fatty acid composition, we performed integrative analysis of fatty acid profile and transcriptome pattern in five types of beef cuts (tenderloin, longissimus dorsi, rump, neck, and chuck) from six adult beef cattle. We evaluated the correlation between gene expression patterns and fatty acids, assessed the co-expressed module and candidate genes related to important fatty acid composition. Finally, we performed protein-protein interaction (PPI) and transcription factor (TF) prediction analysis.

## 2. Materials and Methods

### 2.1. Ethics Statement

All animals were treated following the guidelines for experimental animals which were established by the Council of China. Tissue samples from beef cattle were collected with the approval of the Science Research Department of the Institute of Animal Science, Chinese Academy of Agricultural Sciences under IAS2020-48.

### 2.2. Sample Collection

Six male Chinese Simmental beef cattle were originated from Ulgai, Xilingol League, Inner Mongolia of China. All individuals were weaned at six months of age and then fattened to 24 months of age under the same feeding and management conditions. The cattle were moved to Inner Mongolia ZhongAo Food Co., Ltd (Chifeng City, Inner Mongolia Autonomous Region, China) for slaughter. During the period of slaughtering, samples from chuck, neck, rump, tenderloin, and longissimus dorsi were collected and soaked in RNAlater (Qiagen, Hilden, Germany) and snap frozen in liquid nitrogen. Meanwhile, meat samples from chuck, neck, rump, tenderloin, and longissimus dorsi were collected and stored for 48 h, and then samples were vacuum packed and chilled at −80 °C. Approximately 10 g of fresh samples were used for subsequent analyses.

### 2.3. Fatty Acid Determination

The fatty acid methyl ester of meat was prepared according to the GB/T 5009.168-2016 Standard. Brief, 0.5 g freeze-dried meat in powder state was added to a hydrolysis tube with 2 mL of n-hexane. Four milliliters of chloroacetyl methanol solution (chloroacetyl methanol = 1 + 10) were added and then the samples agitated 1 min. Subsequently, the samples were placed in a constant-temperature water bath at 80 °C, and agitated from water bath every 20 min, up to 2 h and rapidly cooled to room temperature. 4 mL of K_2_CO_3_ were added for saponification and methyl esterification. This mixture was transferred to a 10 mL glass centrifuge tube, and centrifuged at 1300 rpm, for 5 min at 4 °C. Fatty acid methyl esters were quantified with a gas chromatography (GC-2014 CAFsc, Shimadzu Scientific Instruments). The column oven temperature was held at 140 °C for 3 min, then increased to 220 °C at a rate of 4 °C min ^−1^, and held at 220 °C for 5 min, and subsequently increased to 230 °C, and held at 230 °C for 25 min. One μL sample was analyzed. Fatty acid composition was identified by comparison of retention time of methyl esters of the samples with the GB/T 5009.168-2016 and commercial standard for 37 fatty acids Supelco TM Component FAME Mix (cat 18919, Supelco, Bellefonte, PA, USA). Fatty acid composition was measured based on a gravimetric basis (g/100 g). In addition, fatty acid composition was quantified as the percentage of total fatty acids as described in previous study [6].

### 2.4. RNA Extraction, Library Preparation, and Sequencing

The total RNA was extracted from the 30 muscle tissue samples by using Trizol method, and subjected to quality control by the NanoDrop^®^ 2000 (Thermo, Carlsbad, CA, USA) and treated with DNase I (RNase-free) following the manufacturer’s instructions. RNA purity and integrity were assessed by agarose gel electrophoresis. RNA concentration was measured using a Qubit RNA BR Assay Kit (Q10210; Thermo Fisher Scientific, Carlsbad, CA, USA) and RNA integrity was detected by the Agilent Bioanalyzer 2100 system (Agilent Technologies, CA, USA). mRNA libraries were prepared following the TruSeq Stranded library protocols using 5 μg of total RNA, and sequenced on the Illumina HiSeq 2500 sequencing platform (Illumina, San Diego, CA, USA). The 250–300 bp fragment size was selected with AMPure XP beads and performed with PCR enrichment of the fragment for the library. To ensure quality of the library, PCR products were purified (AMPure XP beads) and quality was assessed on the Agilent Bioanalyzer 2100 system. At last, ~6 G raw data was generated per sample from the Illumina Nova Seq 6000 system.

### 2.5. Data Quality Control and Processing

The raw paired-end data from the muscle tissues were trimmed and the clean data with high quality reads were obtained using FASTP software with default parameters [11]. The index of the ARS-UCD1.2 reference genome (Bethesda, MD, USA) was built using the HISAT2-build software (v.2.1.0, HISAT2, Maryland, USA) [12]. The clean data for each sample were mapped to the reference genome using HISAT2 [12]. SAMtools (v.1.9, SAMtools, Harvard, Cambridge, MA, USA) was used to sort and convert the files from SAM to BAM format [13]. The transcripts and expressed genes were assembled and quantified by STRINGTIE (v.2.14, STRINGTIE, Maryland, USA) [14]. After assembling each dataset, the transcripts.gtf from all samples were constructed by the merge function implemented in STRINGTIE. The transcripts abundances and gene expression levels were re-estimated based on transcripts.gtf file using STRINGTIE software. The expression levels of transcripts and genes in each sample were estimated using read counts and fragments per kilobase of transcript per million mapped reads (FPKM).

### 2.6. Gene Expression Pattern across Tissues

To explore the gene expression patterns of five types of beef cuts, we first calculated the gene expression (FPKM > 1) in six individuals. The expression level of each gene was corrected by log-transformation (log_2_ (FPKM + 1)). To detect intrinsic repeatability and outliers of the muscle samples, the five types of beef cut samples were clustered and visualized by principal component analysis (PCA) using prcomp function in R (v.4.1.1, R, Vienna, Austria). In addition, hierarchical clustering of these samples was implemented using the Pheatmap package.

### 2.7. Analysis and Annotation of Differentially Expressed Genes

To identify the DEGs between samples, we assessed the number of read counts and estimated the expression level of each transcript and gene. DEGs were detected using Deseq2 package. The selected criteria for DEGs were as follows: the absolute log_2_ Fold Change value > 1.5, and the *p*-value < 0.05. Intersection sets of DEGs between five type of beef cuts were visualized using UpSetR package [15]. Volcano plots implemented in the ggpubr package were used to display DEGs. We finally performed a functional GO term enrichment analysis of DEGs using clusterProfiler package [16].

### 2.8. Weighted Gene Co-Expression Network Construct

We applied a weighted gene co-expression network analysis (WGCNA) to construct a co-expression network for different beef cuts [17]. Briefly, a total of 4511 genes with FPKM >1 was used for module constructions. The hierarchical cluster analysis on all tissues was carried out using the *hclust* function. The soft threshold (β) was calculated based on the principle of scale-free distribution, and the β = 5 value was determined as the appropriate soft threshold power value. To investigate the relationship between the module and fatty acid composition, we assessed the relevance of co-expression modules with 26 fatty acid compositions and seven fatty acid groups using Pearson’s correlation between the module eigengene and target trait. We performed functional enrichment analyses for fatty acid composition related module genes using the Database for Annotation, Visualization, and Integrated Discovery (DAVID) v6.8 with Bonferroni’s correction [18].

### 2.9. Analysis PPIs and TF of Fatty Acid Composition Candidate Genes

To explore the protein interaction for fatty acid candidate genes, the protein-protein interaction (PPI) network was constructed using the STRING (v11, STRING, Zurich, Switzerland) database. Based on the STRING analysis, a network diagram of the fatty acid candidate genes was drawn using Cytoscape software (v3.7.1, Cytoscape, Seattle, Washington, USA) [19]. In addition, the fatty acid candidate genes were used to predict transcription factor-binding sites (TFs) using iRegulon tool (v.1.3, iRegulon, Leuven, Belgium) [20]. The regulation network of TFs and co-expressed genes was constructed using Cytoscape software. The size of TFs was displayed based on their Normalized enrichment score (NES).

### 2.10. Real-Time Quantitative PCR (RT-qPCR) Analysis

To validate the expression of candidate genes related to fatty acid composition, 10 candidate genes were randomly selected for qRT-PCR analyses using the QuantStudio 7 Flex real-time PCR System (Life Technologies, Carlsbad, CA, USA). Total RNA from the tenderloin, longissimus dorsi, rump, neck, and chuck samples were extracted using Trizol method, and quality was checked by NanoDrop^®^ 2000 (Thermo, CA, USA) and treated with DNase I (RNase-free). The cDNA was synthesized by reverse candidate genes using Prime Script™ RT Reagent kit with gDNA Eraser (Takara, Dalian, China). RT-qPCR primers for candidate genes were designed using the Primer Premier 5 (Supplementary Table S1) and synthesized by Sangon Biotech (Sangon, Shanghai, China). The 10 μL RT-qPCR reaction contained 0.5 μL cDNA, 0.4 μL of each primer (F/R), 5 μL 2 × KAPA SYBR^®^ FAST (KAPABiosystems, Wilmington, MA, USA), 0.2 μL Rox Low, 3.5 μL H_2_O. The amplification cycle was as follows: initial denaturation for 3 min at 95 °C for 1 cycle, followed by 40 cycles at 95 °C for 2 s, 60 °C for 20 s. The 2^−ΔΔCt^ method was used to transform Ct values, and the expression levels of muscle samples from five beef cuts were compared with the basal value using the nonparametric Kruskal–Wallis test and Nemenyi test. Samples from three independent experiments were assayed. The *GAPDH* gene was used as an endogenous control to estimate gene expression levels.

## 3. Results

### 3.1. Difference of Fatty Acids and Fatty Acid Groups in Different Beef Cuts

**Figure 1:**
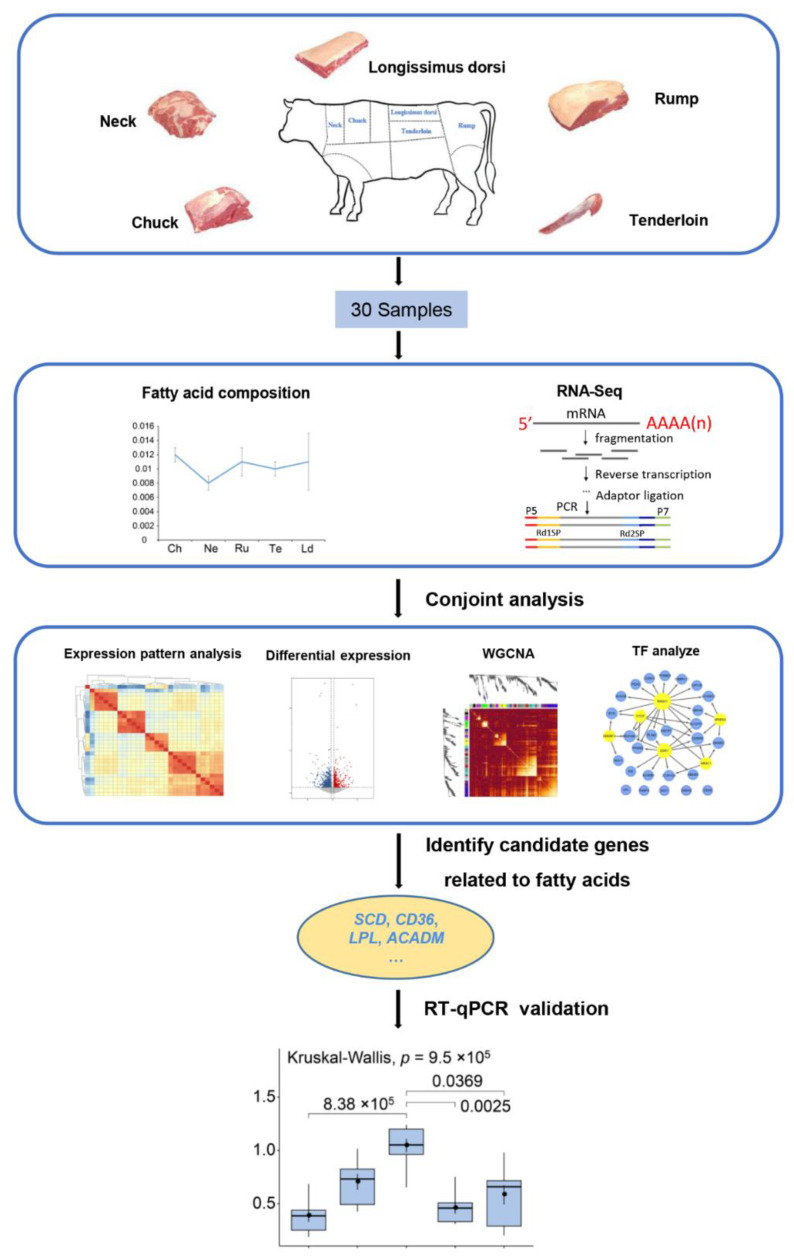
Schematic view of the present study. We used six male Chinese Simmental beef cattle across five beef cuts to study the difference in fatty acids through multifaceted analyses (expression pattern analysis, differentially expressed gene analysis, co-expression analysis, TF analysis). The figure at the top shows the beef cut locations in this study. The figure at the bottom shows validation of the expression levels of candidate genes related to fatty acid traits using RT-qPCR. In the box plot: the maximum value (top of the line), the minimum value (low end of the line), the median (black point), the upper quartile (the upper border of the rectangle), the lower quartile (the bottom border of the rectangle), and the invalid data (out-of-line outliers).

To explore the fatty acid changes between different beef cuts, we collected five representative beef cuts including tenderloin, longissimus dorsi, rump, neck, and chuck cuts from adult beef cattle. The fatty acid compositions from five different types of beef cuts were estimated and RNA-Seq data were generated from 30 samples. Then, we analyzed gene expression patterns, differential gene expression, and co-expression networks. We finally validated the mRNA levels of ten candidate genes by RT-qPCR. The workflow of this study was shown in Figure 1.

We assessed the differences in fatty acid composition by measuring the fatty acid spectrum in five representative beef cuts. Gas chromatography analysis was performed to characterize the fatty acid spectrum of five types of beef cuts, and the content of 26 fatty acid compositions and seven fatty acid groups were estimated according to the GB/T 5009.168-2016 Standard (Supplementary Table S2). Each fatty acid was quantified as a percentage of total fatty acids. The palmitic acid (C16:0), oleic acid (C18:1n9c), and stearic acid (C18:0) were the main fatty acid compositions in muscle (Supplementary Table S3). Among these beef cuts, we observed obvious differences for different fatty acid compositions (Supplementary Figure S1). The content of the UFA group, including MUFA (C16:1 C18:1n9c, C20:1) and PUFA (C18:2n6c, and C18:3n3) in the tenderloin were higher than other beef cuts. Notably, the tenderloin has high content of fatty acids of n3 and n9 groups, followed by the longissimus dorsi (Supplementary Figure S1).

### 3.2. Summary Statistics of Sequencing Dataset

To identify the potential genes involved in the regulation of fatty acid composition in muscle, we performed RNA-seq analysis with six biological replicates for tenderloin, longissimus dorsi, rump, neck, and chuck cuts. In total, approximately 682 million raw paired-end reads (~204 GB) were generated, with an average of 22 million reads per sample (Supplementary Dataset S1). After rigorous quality control (see Methods), the average reads effective rate was ~96.97% (range from 95.49 to 97.65%) among 30 beef cuts samples. In this study, a total of 198 GB high-quality data was mapped to the reference genome (ARS-UCD1.2), using the HISAT2 software with the default setting [12]. The average reads mapping rate was ~95.27%, ranging from 94.23 to 97.02%. Detailed information about reads mapping information and summary statistics for each sample were shown in the Supplementary Dataset S2. The gene expression level was quantified with FPKM value using StringTie software [14], and 15,701 genes were obtained. Considering the influence of biological and technological confounding factors, genes with FPKM > 1 in the six biological replicate samples were retained and 4511(28.7%) genes were detected with the reliable expression levels (Supplementary Dataset S2).

### 3.3. Gene Expression Profile across Tissues

**Figure 2:**
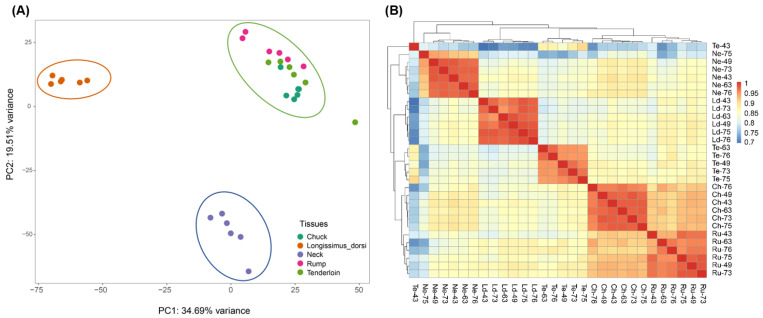
Gene expression profile among the five beef cuts. (**A**) Principal component analysis for all tissue types based on log_2_ (FPKM + 1) corrected expression data. (**B**) Unbiased hierarchical clustering heat map based on Pearson’s correlation coefficient for 4511 genes. The ordinate and coordinates are both tissue names. Ld, Ch, Ru, Te, and Ne are the abbreviations of longissimus dorsi, chuck, rump, tenderloin, and neck tissues, respectively. Color intensity indicates the correlation between tissues, red indicates high correlations (1), and blue indicates low correlations (0.7).

To explore the transcriptome changes and biological clustering across five different beef cuts including tenderloin, longissimus dorsi, rump, neck, and chuck cuts, we firstly performed principal component analysis (PCA) on gene expression in 30 samples. The PCA analysis for five types of muscle showed that 54.2% of the variance can be explained by the first two principal components, accounting for 34.69%, and 19.51% of the variance, respectively (Figure 2A). Basically, five types of beef cuts were clearly clustered into three separate groups (i.e., neck, longissimus dorsi, as well as tenderloin, rump, and chuck), which indicated the different transcriptome profiles between these five beef cuts (Figure 2A).

The hierarchical clustering analysis was conducted to investigate the transcriptome profile within different beef cuts based on inter-tissues correlations. The heatmap depicted the correlations between samples, where deeper red represents the higher correlation and deeper blue represents lower correlation. The 30 samples were displayed as columns and rows, and classified by subtypes. Our results showed distinct clustering of these five types of beef cuts (Figure 2B). Chuck and rump cuts had a similar tendency and clustered together. Notably, tenderloin cut was aggregated into a cluster, and separated from longissimus dorsi and neck cuts. These results suggested diverse transcriptome changes in five types of beef cuts.

### 3.4. Differential Expression Analysis between Five Different Beef Cuts

**Figure 3:**
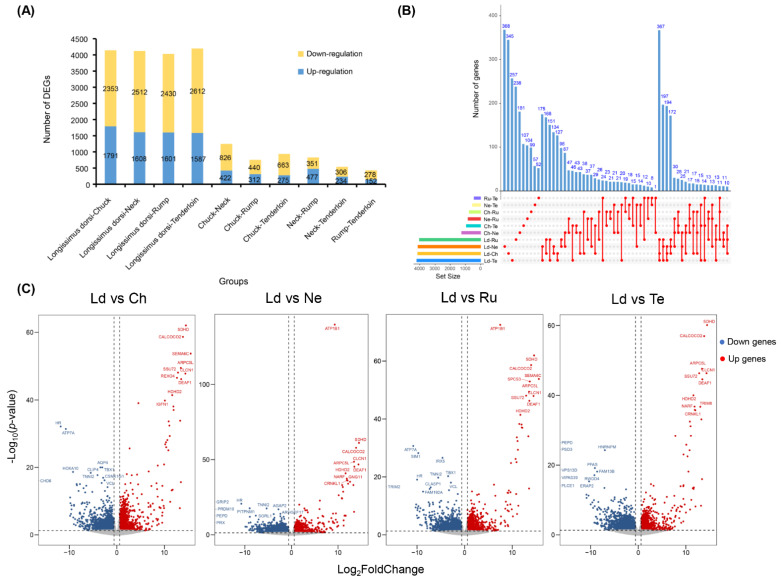
Identification of DEGs. (**A**) The number of up-regulated and down-regulated DEGs identified in each group. (**B**) Venn diagram of unique and shared DEGs in the ten groups. (**C**) The Volcano plot from Ld vs. Ch, Ld vs. Ne, Ld vs. Ru, and Ld vs. Te groups, respectively, the other comparisons are shown in Supplementary Figure S2. The abscissa is log_2_ (Fold Change) value, and the ordinate is −log_10_ (*p*-value). Blue nodes represent down-regulated genes, red nodes represent up-regulated genes.

To explore potential difference of transcriptomic expression for fatty acid compositions among beef cuts, we evaluated the transcriptome changes of five beef cuts using RNA-sequencing and detected DEGs between them based on read counts (Figure 3A). We observed the number of DEGs among ten groups ranges from 430 in the rump and tenderloin group (152 up-regulated and 278 down-regulated genes) to 4199 in the longissimus dorsi and tenderloin group (1587 up-regulated and 2612 down-regulated genes) (Supplementary Table S4). In addition, we obtained a total of 7545 DEGs among 10 differential beef cut groups, the unique DEGs among ten groups ranged from 52 in the rump and tenderloin group to 368 in the longissimus dorsi and neck (Figure 3B). Meanwhile, the up-regulated and down-regulated genes were showed in the ten groups using the Volcano plot, respectively (Figure 3C, Supplementary Figure S2), and the fold changes of these genes across different groups were presented in the Supplementary Dataset S3.

To assess the functional contribution of DEGs, we performed a gene annotation analysis using DAVID v6.8. DEGs identified across 10 pairwise comparisons were enriched in biological process (BP) terms including peptide metabolic/biosynthetic process (GO: 0006518/GO: 0043043), translation (GO: 0006412), mitochondrial gene expression/translation (GO: 0140053/GO: 0032543); cellular component (CC) terms including mitochondrial protein complex (GO: 0098798), respiratory chain complex (GO: 0098803), oxidoreductase complex (GO: 1990204); and molecular function (MF) terms including structural constituent of ribosome (GO: 0003735) and NADH dehydrogenase activity (GO: 0003954) (Supplementary Table S5). Functional enrichment analysis suggested that the identified DEGs were involved in oxidative phosphorylation (bta00190), thermogenesis (bta04714), calcium ion signaling pathways (bta04020), ribosomes (bta03010), and fatty acid metabolism pathways (bta01212) (Supplementary Table S6).

### 3.5. Gene Co-Expression Analysis Reveals Fatty Acid Specific Modules in Muscles

**Figure 4:**
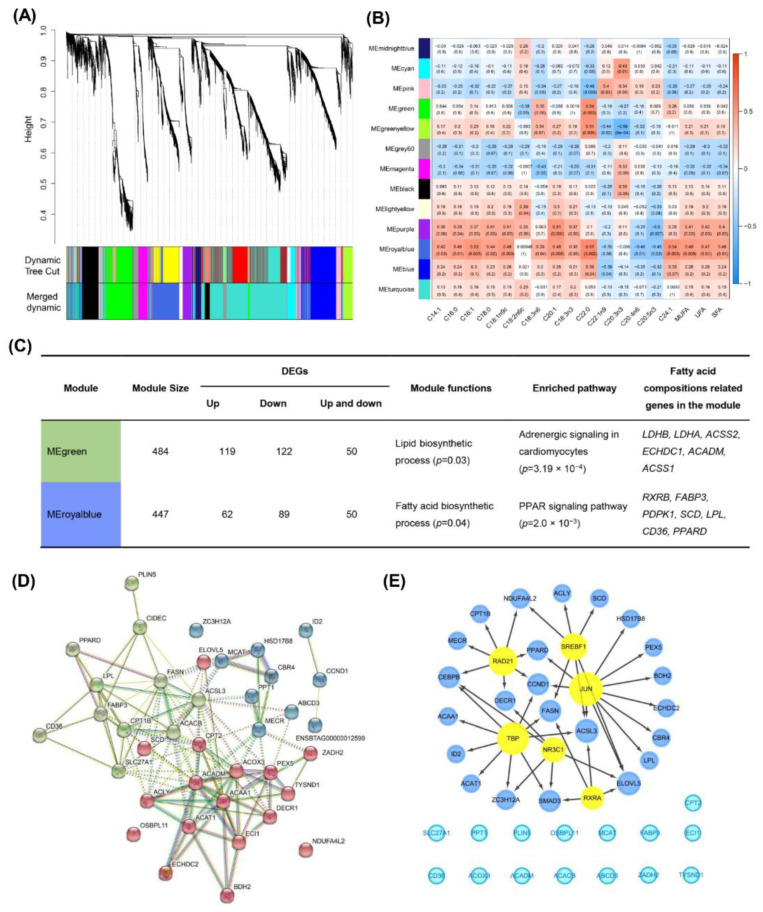
Co-expression network detection in five beef cuts. (**A**) 4511 genes-enriched modules in the co-expression network. Functional modules are represented in different colors. Each major branch in the figure represents a color-coded module that contains a group of highly connected genes. (**B**) Heatmap between 13 modules and 18 fatty acid composition. The complete heatmap of 13 modules and 33 fatty acid traits is shown in Figure S6. Boxes contain Pearson correlation coefficients and their associated *p* values. Red color indicates that the given organization has a strong positive correlation to all other organizations. Blue color indicates that the given organization has a strong negative correlation to all other organizations. (**C**) Putative functions and related fatty acid composition core genes in the fatty acid-related modules. (**D**) The PPI network of 38 genes related to fatty acid traits. (**E**) TF analysis of 38 genes related to fatty acid traits. Yellow nodes represent transcription factors and blue nodes represent the regulatory target genes.

To explore the biological relationships and potential functions of core driver genes related to fatty acid composition and fatty acid groups, the WGCNA was performed on the expression levels of 4511 genes and the content of 26 fatty acid compositions and seven fatty acid groups. We performed a clustering dendrogram on 30 samples based on their Euclidean distances, and finally kept 28 samples for subsequent network analysis (Supplementary Figure S3). A soft threshold power (β = 5) was selected based on scale independence and mean connectivity (Supplementary Figure S4), and k was negatively correlated with p(k) (correlation coefficient was 0.89), indicating that the selected β value can effectively establish a scale-free network (Supplementary Figure S5). Using co-expression network analysis, 13 modules were identified, and the number of genes for each module ranged from 59 to 1443 (Figure 4A, Supplementary Table S7). To identify fatty acid composition specific modules, we calculated the correlation coefficients between 13 modules and fatty acid composition. Our result showed that the MEgreen module was significantly correlated with two fatty acid composition (C22: 0, C22:2) (Supplementary Figure S6), and the MEroyalblue module showed significant correlations with 20 fatty acid compositions (e.g., C14:1, C16:1, C18:1n9c, C18:3n6, C24:1) (Figure 4B, Supplementary Figure S6). The functional annotations of genes in fatty acid composition specific modules indicated that MEgreen module was involved in lipid biosynthetic process (GO:0008610) and lipid particle (GO:0005811). Notably, the genes included in MEroyalblue module were involved in the regulation of fat cell differentiation (GO:0045598) and fatty acid biosynthetic process (GO:0006633) (Figure 4C). Moreover, pathway enrichment analyses showed that the genes in MEgreen module were involved in the regulation of adrenergic signaling in cardiomyocytes (bta00640). At the same time, the genes in MEroyalblue module were mainly involved in the regulation of PPAR signaling pathways (bta03320) (Figure 4C).

Additionally, we identified 38 candidate genes related to fatty acid composition from the 13 modules (Supplementary Dataset S4). We constructed a protein interaction network for 38 candidate genes based on the string database (v11, STRING, Zurich, Switzerland). We observed these genes were mainly involved in biological processes, including fatty acid metabolic process (GO:0006631), lipid catabolic process (GO:0016042), fatty acid biosynthetic process (GO:0006633), and long-chain fatty acids import into cell (GO:0044539) (Supplementary Table S8). Pathway enrichment showed that genes were mainly involved in PPAR signaling pathway (bta03320), fatty acid degradation (bta00071), and AMPK signaling pathway (bta04152) (Supplementary Table S8). We also found that fatty acid-related genes clustered into three categories, based on K-means clustering (Figure 4D). The *PEX5*, *ACOX3*, *ACAA1*, and *ACADM* genes were at the core of the protein network interaction (denoted by red-colored nodes). The *FABP3*, *LPL*, *FASN*, and *ACACB* genes were at the core of the protein network interaction (denoted by green-colored nodes). The *MECR* gene was at the core of the protein network interaction (denoted by blue-colored nodes) (Figure 4D). To identify the regulatory factors, we performed TF analysis on 38 candidate genes related to fatty acid composition. A total of six TFs were identified, including SREBF1 (NES = 5.960), NR3C1 (NES = 3.863), RAD21 (NES = 3.511), TBP (NES = 3.271), RXRA (NES = 3.215), and JUN (NES = 3.108), which were used to construct regulatory networks (Figure 4E).

### 3.6. Differently Expressed Candidate Genes Related to Fatty Acid Composition across Different Beef Cuts

**Figure 5:**
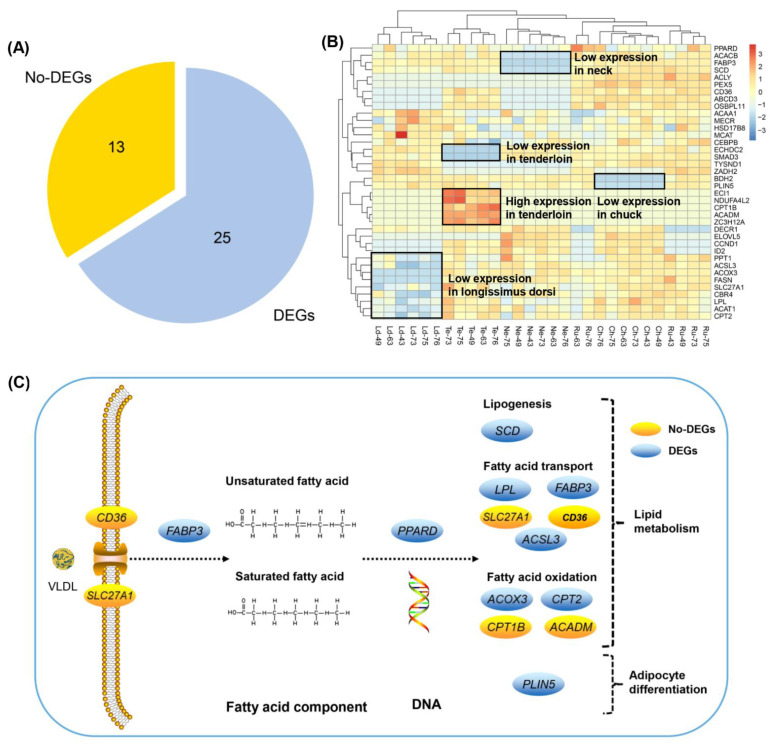
Genes involved in fatty acid biological processes. (**A**) The pie chart shows the number of DEGs and no-DEGs in the 38 candidate genes related to fatty acid traits. (**B**) Heat maps of 38 candidate genes in five beef cuts. (**C**) The candidate genes involved in lipid metabolism and adipocyte differentiation pathway. DEGs are represented by blue. Black dashed lines indicate more than one step in the path.

The primary of this study was to investigate the expression differences involved in fatty acid composition changes between beef cuts. Therefore, we focused on the expression regulation of candidate genes that were involved in fatty acid synthesis, fatty acid transport, fatty acid degradation, and fatty acid beta-oxidation processes in different beef cuts. We obtained a total of 38 candidate genes related to fatty acid composition, of which 25 genes were differentially expressed in different beef cuts (Figure 5A). Meanwhile, we observed the expression levels of 38 candidate genes varied among five cuts (Figure 5B). We found that a cluster of genes (e.g., *ECI1*, *NDUFA4L2*, *CPT1B*, *ACADM*, and *ZC3H12A*) were significantly highly expressed in the tenderloin, while two genes (*ECHDC2*, *SMAD3*) were low expressed in the tenderloin. In addition, we observed some genes with low expression in the longissimus dorsi (e.g., *ACOX3*, *FASN*, *SLC27A1*), neck (*ACACB*, *FABP3*, *SCD*), chuck (*BFH2*, *PLIN5*), and rump (*DECR1*) cuts, respectively. The expression levels of these genes were different, which may affect the formation of fatty acid composition in different beef cuts. Notably, we found 14 candidate genes which were enriched in the lipid metabolism and adipocyte differentiation pathways of beef cattle (Figure 5C). *SCD* gene was involved in lipogenesis. *CD36*, *SLC27A1*, *LPL*, *FABP3*, and *ACSL3* play important roles in fatty acid transport. *ACOX3*, *CPT2*, *CPT1B*, and *ACADM* regulate fatty acid oxidation. In addition, the DEG, *PLIN5*, may be important for adipocyte differentiation in beef cattle.

### 3.7. Validating Expression Profiles of Candidate Genes

**Figure 6:**
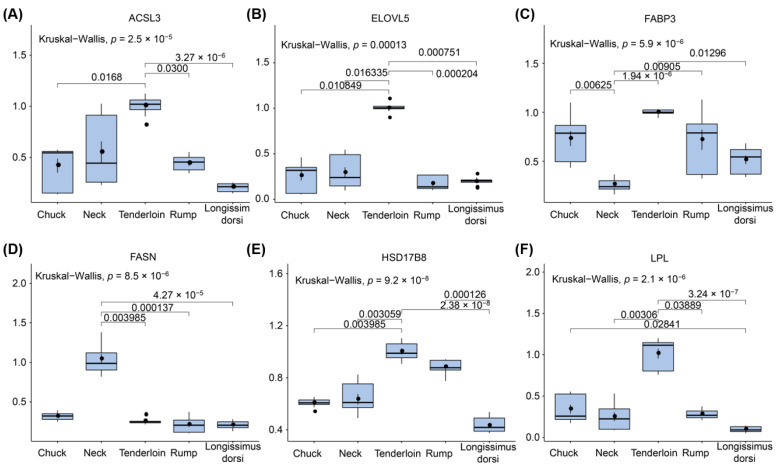
Validation of the expression levels of candidate genes related to fatty acid traits using RT-qPCR. Boxplots show the mRNA expression levels of six candidate genes randomly selected in five types of beef cuts in Chinese Simmental beef cattle (*n* = 6). (**A**) *ACSL3*, (**B**) *ELOVL5*, (**C**) *FABP3*, (**D**) *FASN*, (**E**) *HSD17B8*, (**F**) *LPL*. The ordinate is the relative expression level value, and the abscissa is the name of the bovine tissue sample. In the box plot: the maximum value (top of the line), the minimum value (low end of the line), the median (black point), the upper quartile (the upper border of the rectangle), the lower quartile (the bottom border of the rectangle), and the invalid data (out-of-line outliers).

To validate the expression profile of candidate genes derived from by RNA-seq, we performed RT-qPCR on five beef cuts from the same samples. Ten important candidate genes including *ACSL3*, *ELOVL5*, *FABP3*, *FASN*, *HSD17B8*, *LPL*, *PEX5*, *PLIN5*, *PPARD*, and *SCD* were selection for subsequently analysis. Consistent with gene expression changes from RNA sequencing, we found significant differences among the gene expression values among different beef cuts (Figure 6, Supplementary Figure S7). Moreover, positive correlations between candidate gene expressions and beef cuts were demonstrated in all boxplots. There were significant differences in the expression of candidate genes between tenderloin and longissimus dorsi, for instance, *ACSL3* (*p* = 3.27 × 10^−6^), *ELOVL5* (*p* = 7.51 × 10^−4^), *FABP3* (*p* = 1.30 × 10^−2^), *HSD17B8* (*p* = 2.38 × 10^−7^), *LPL* (*p* = 3.24 × 10^−7^), *PEX5* (*p* = 3.69 × 10^−2^), *PLIN5* (*p* = 1.37 × 10^−2^), *PPARD* (*p* = 1.08 × 10^−3^), and *SCD* (*p* = 3.51 × 10^−2^). *ACSL3*, *ELOVL5*, *PEX5* genes showed different expression between tenderloin and rump, as well as between tenderloin and chuck. Meanwhile, significant differences were observed between tenderloin and neck for *ELOVL5* (*p* = 1.63 × 10^−2^), *FABP3* (*p* = 1.94 × 10^−6^), *FASN* (*p* = 3.99 × 10^−3^), *HSD17B8* (*p* = 3.06 × 10^−3^), *LPL* (*p* = 3.06 × 10^−3^), *PLIN5* (*p* = 4.84 × 10^−3^), and *PPARD* (*p* = 6.00 × 10^−5^) (Figure 6, Supplementary Figure S7). Our RT-qPCR analyses confirmed that candidate genes for fatty acid composition were differentially expressed across beef cuts and further supported our RNA-seq results.

## 4. Discussion

Fatty acid composition is an important indicator of the nutritional value and affect meat quality. In the present study, we reported the fatty acid composition changes among different types of beef cuts. Moreover, we carried out a comprehensive transcriptomic analysis of candidate genes and their expression difference underlying fatty acid composition in beef cattle.

### 4.1. Detection of Fatty Acid Composition in Different Beef Cuts

Fatty acid metabolism was a complex and dynamic process, and fatty acid composition can be affected by various factors such as age, sex, breed, beef cuts in beef cattle [9,21]. In the current study, we detected 26 fatty acid compositions and seven fatty acid groups in beef cattle and analyzed the differences in fatty acids in tenderloin, longissimus dorsi, rump, neck, and chuck cuts. We observed the content of UFA in tenderloin were higher than other beef cuts. Previous studies have demonstrated that different muscles have intrinsic fiber types, resulting in distinct meat properties and metabolic patterns [22]. Tenderloin tissue has a higher proportion of type I fibers, and more lipid, protein, and myoglobin oxidation compared to other muscle tissues [23,24,25]. Compared with the ribeye muscle, the rump contains lower SFA and MUFA, but has a higher proportion of polyunsaturated fatty acids without exceeding the critical limit, thus rump have higher nutritional indicators [26]. Our result confirmed the tenderloin cut with higher nutritional value was enriched with a high proportion of UFA, followed by the longissimus dorsi cut.

### 4.2. Candidate Genes Involved in Fatty Acid Transport and Lipogenesis

Blood circulation is one of the main sources of fatty acids. The fatty acid can be used to synthesize triacylglycerols and form lipid droplets when its uptake exceeds the fatty acid oxidation in muscle [27,28]. Several genes (e.g., *CD36*, *SLC27A1*, *FABP3*, *LPL*, and *ACSL3*) have been reported to be involved in fatty acid transport. For instance, *CD36* (cluster of differentiation 36) is a scavenger receptor that plays a key role in the absorption of long-chain fatty acids (FAs) and contributes to lipid accumulation and metabolic functions under excessive fat [29,30]. *SLC27A1* is a regulated fatty acid transporter involved in regulating the long chain fatty acid uptake into cells [31]. As members of the fatty acid binding proteins (FABP) family, *FABP3* is considered to be a lipid partner, which may regulate the solubility, fluidity and utilization of fatty acids [32]. *LPL* (lipoprotein lipase) is a key rate-limiting enzyme that regulates the transport of chylomicrons and very low-density lipoprotein (VLDL) in the hydrolyzed triglyceride (TG) cycle [33,34]. *ACSL3* is a member of the long-chain family of acyl-CoA synthase, which activates free fatty acids into fatty acid acyl-CoA to synthesize glycerophospholipids [35]. For fatty acid transport, *CD36*, *SLC27A1* and *LPL* regulated the extracellular transport of fatty acids from capillaries to cytoplasm, while *FABP3* was involved in the transport of fatty acids from cytoplasm to organelle membranes [36]. In addition, stearoyl-CoA desaturase (SCD) is a key enzyme in lipogenesis, regulating and catalyzing the conversion of SFA to MUFA [37]. Our transcriptome analysis showed that four out of six genes were differently expressed among five different type of beef cuts (Figure 5). We observed three genes (*ACSL3*, *LPL*, and *SCD*) were down-regulated between the longissimus dorsi and tenderloin, and the log_2_ (fold change) value of *LPL* was −1.31. *FABP3* was up-regulated between chuck and neck, and the log_2_ (fold change) value was 2.29 (Supplementary Dataset S3). Our results indicated that differentially expressed candidate genes for fatty acid transport may affect the formation of fatty acid composition. In addition, fatty acid candidate genes may be regulated by TFs, which affect fatty acid synthesis and transport processes. SREBF1 was involved in regulating lipid homeostasis [38]. TBP, along with fatty acid synthase (FASN), was involved in mTOR signaling pathway [39]. Long-chain fatty acid can be combined with RXRA as a ligand and was essential for hematopoietic system stress [40].

### 4.3. Candidate Genes Involved in Fatty Acid Oxidation and Adipocyte Differentiation

Fatty acid degradation involves lipolysis, long-chain fatty acid activation, and fatty acid beta-oxidation [41]. Four candidate genes (i.e., *ACOX3*, *CPT2*, *CPT1B*, and *ACADM*) were involved in regulating fatty acid beta-oxidation in this study (Figure 5). *ACOX3* (Acyl-Coenzyme A oxidase 3, pristanoyl), was highly expressed in muscle tissues from tenderloin and neck, the log_2_ (fold change) value were 1.12 and 1.16, respectively, and this gene was involved in the desaturation of 2-methyl branched fatty acids in peroxisomes [42]. As an essential factor in mitochondrial long chain fatty acid beta-oxidation, *CPT2* (Carnitine Palmitoyltransferase 2) regulated the process of long-chain fatty acids entering the mitochondria and combined with coenzyme A to metabolize and produce energy [43]. *CPT1B* (Carnitine Palmitoyltransferase 1B) regulates fatty acid transport in fatty acid beta-oxidation [44]. *ACADM* (acyl-CoA dehydrogenase medium chain) encodes medium-chain acyl-CoA dehydrogenase, functionally catalyzes the first step of mitochondrial beta-oxidation of medium chain acyl-CoAs [45]. In addition, *PLIN5* (Perilipin 5) encodes lipid droplet protein, maintains the balance of fat production and lipolysis, and regulates fatty acid oxidation [46].

## 5. Conclusions

In summary, we identified 38 candidate genes related to fatty acid composition using differential gene expression and co-expression analysis. Functional annotation suggested that many genes were involved in fatty acid biosynthetic process, fatty acid degradation, and the PPAR signaling pathway. Our findings revealed a subset of candidates involved in fatty acid transport (*CD36*, *SLC27A1*, *FABP3*, *LPL*, and *ACSL3*), lipogenesis (*SCD*), fatty acid oxidation (*ACOX3*, *CPT2*, *CPT1B*, and *ACADM*), and adipocyte differentiation (*PLIN5*). Our results provided some valuable insights into understanding the transcriptomic changes of fatty acid composition in different beef cuts in beef cattle. Moreover, our findings may help to improve the selection for beneficial fatty acid composition in beef cattle. Further analysis using multi-omics can help to comprehensively elucidate the genetic and regulation mechanism of fatty acid composition.

## Data Availability

The datasets used and analyzed during the current study available from the corresponding author on academic request (LYX). The data are not publicly available to preserve the privacy of the data.

## Supplementary Materials

The following are available online at https://www.mdpi.com/article/10.3390/foods11010117/s1, Figure S1: Fatty acid content across five beef cuts (means ± SD, *n* = 6), Figure S2: The volcano plot from Ch vs. Ne, Ch vs. Ru, Ch vs. Te, Ne vs. Ru, Ne vs. Te, and Ru vs. Te groups, respectively, Figure S3: Hierarchical clustering of 30 samples based on the Euclidian distance using average linkage for agglomeration, Figure S4: The determination of the power Beta (β) value is based on the scale free topology criterion (A) and mean connectivity (B) under the weighted gene correlation network analysis (WGCNA) method, Figure S5: Network scale-free topology distribution test based on selected β value, Figure S6: The heatmap of 13 modules and 33 fatty acid traits, Figure S7: Validation of the expression levels of candidate genes related to fatty acid traits using RT-qPCR, Dataset S1. Reads mapping summary of chuck, neck, rump, tenderloin, and longissimus dorsi, Dataset S2. The statistical gene FPKM value in Longissimus dorsi, chuck, neck, rump and tenderloin tissues, Dataset S3. The summarize the up-regulation and down-regulation of differentially expressed genes (DEGs) in Longissimus dorsi, chuck, neck, rump and tenderloin tissues, Dataset S4. Functional description of 38 fatty acid-related genes, Table S1. RT-qPCR primer sequences of candidate genes related to fatty acid traits in the beef cattle Table S2. Relative content of fatty acid components in chuck, neck, rump, tenderloin, and longissimus dorsi, Table S3. Percentage of fatty acid components in chuck, neck, rump, tenderloin, and longissimus dorsi, Table S4. Analysis of differential gene expression between chuck, neck, rump, tenderloin, and longissimus dorsi tissues, Table S5. GO function annotation analysis of differentially expressed genes among different groups, Table S6. KEGG function enrichment analysis of differentially expressed genes among different groups, Table S7. The identified functional modules using dynamic cutting method, Table S8. Functional annotation enrichment analysis of fatty acid-related genes.

## Abbreviations

Longissimus dorsi, Ld; Chuck, Ch; Rump, Ru; Tenderloin, Te; Neck, Ne; Monounsaturated fatty acids, MUFA; Polyunsaturated fatty acids, PUFA; Saturated fatty acid, SFA; Transcription factor, TF; Unsaturated fatty acid, UFA; Saturated fatty acid, SFA; Differentially expressed genes, DEGs; Differentially expressed proteins, DEPs; Protein-protein interaction, PPI; Fragments per kilobase of transcript per million mapped reads, FPKM; Principal component analysis, PCA; Weighted correlation network analysis, WGCNA; Normalized enrichment score, NES; Biological process, BP; Cellular component, CC; Molecular function, MF; Lipid droplets, LD; Very-low-density lipoprotein, VLDL.
