# Supplementary material for: Comparative Transcriptomic Analysis Reveals Diverse Expression Pattern Underlying Fatty Acid Composition among Different Beef Cuts
Source: Foods. 2022 Jan 4;11(1):117. doi: 10.3390/foods11010117 (PMC8750426; doi:10.3390/foods11010117)
Supplement: Supplementary file 1 [file foods-11-00117-s001.zip › Figure S1-S7.pdf]

Supplementary materials

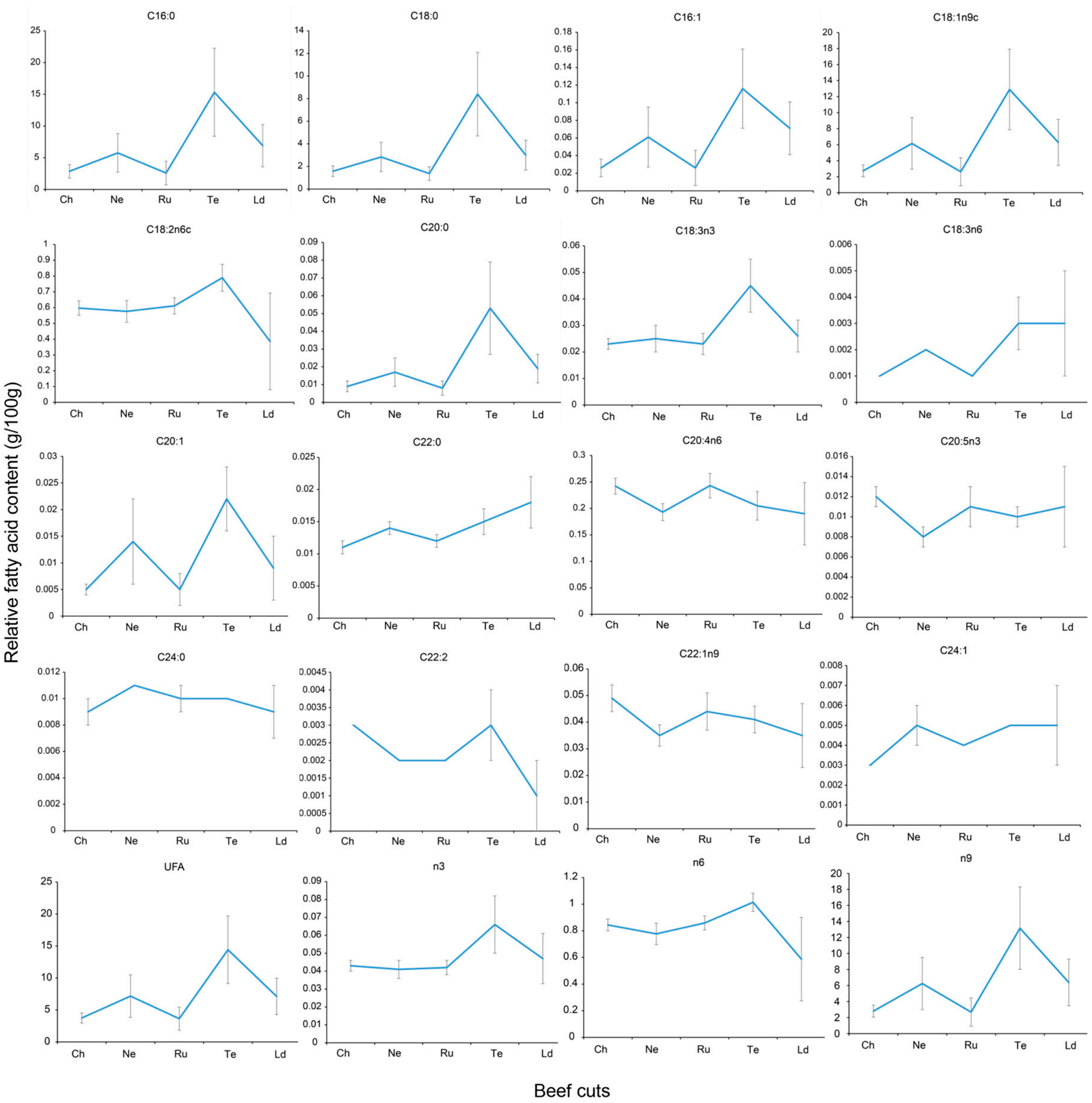

Figure S1: Fatty acid content across five beef cuts (means ± SD, *n* = 6). Ch, Ne, Ru, Te, and Ld are the abbreviations of chuck, neck, rump, tenderloin and longissimus dorsi tissues, respectively.

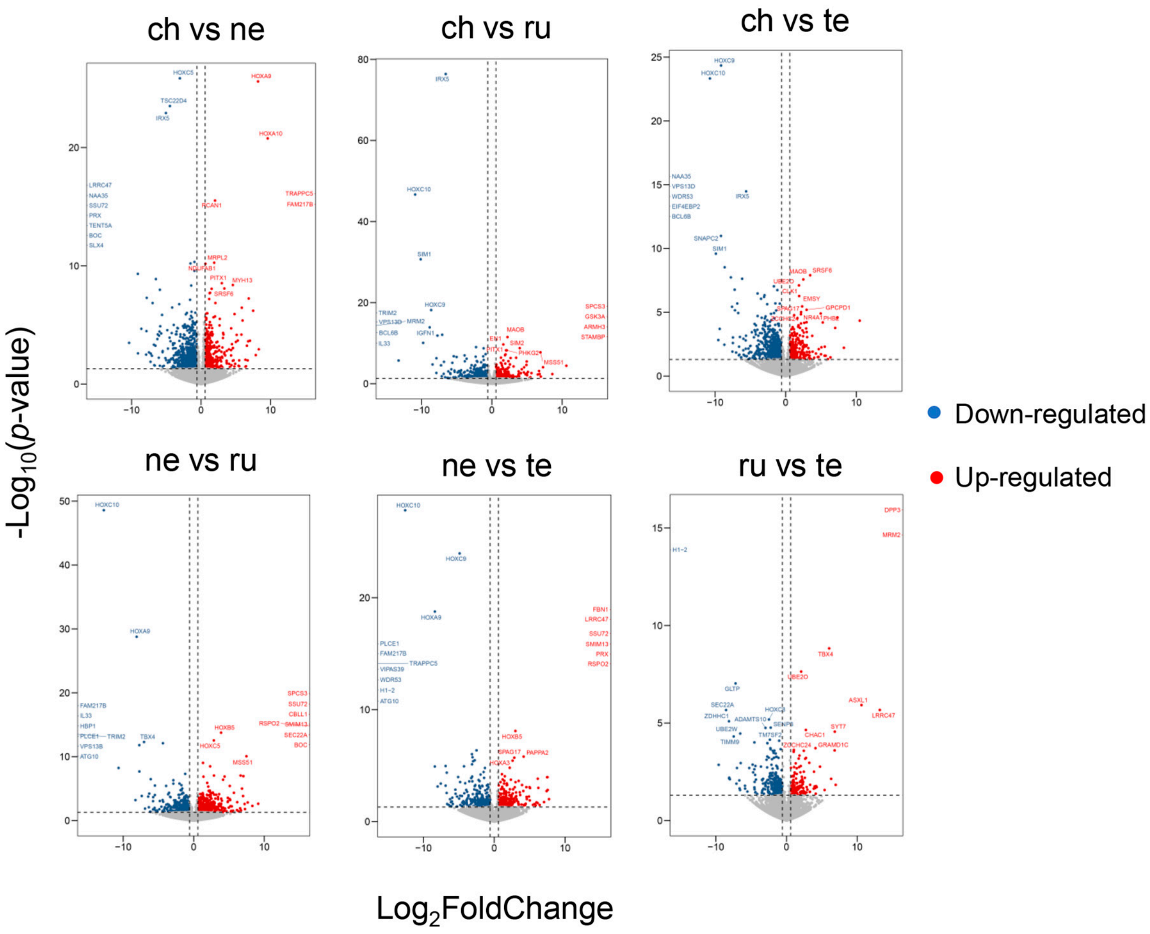

Figure S2: The volcano plot from Ch vs Ne, Ch vs Ru, Ch vs Te, Ne vs Ru, Ne vs Te, and Ru vs Te groups, respectively. Ch, Ne, Ru, Te, and Ld are the abbreviations of chuck, neck, rump, tenderloin and longissimus dorsi tissues, respectively.

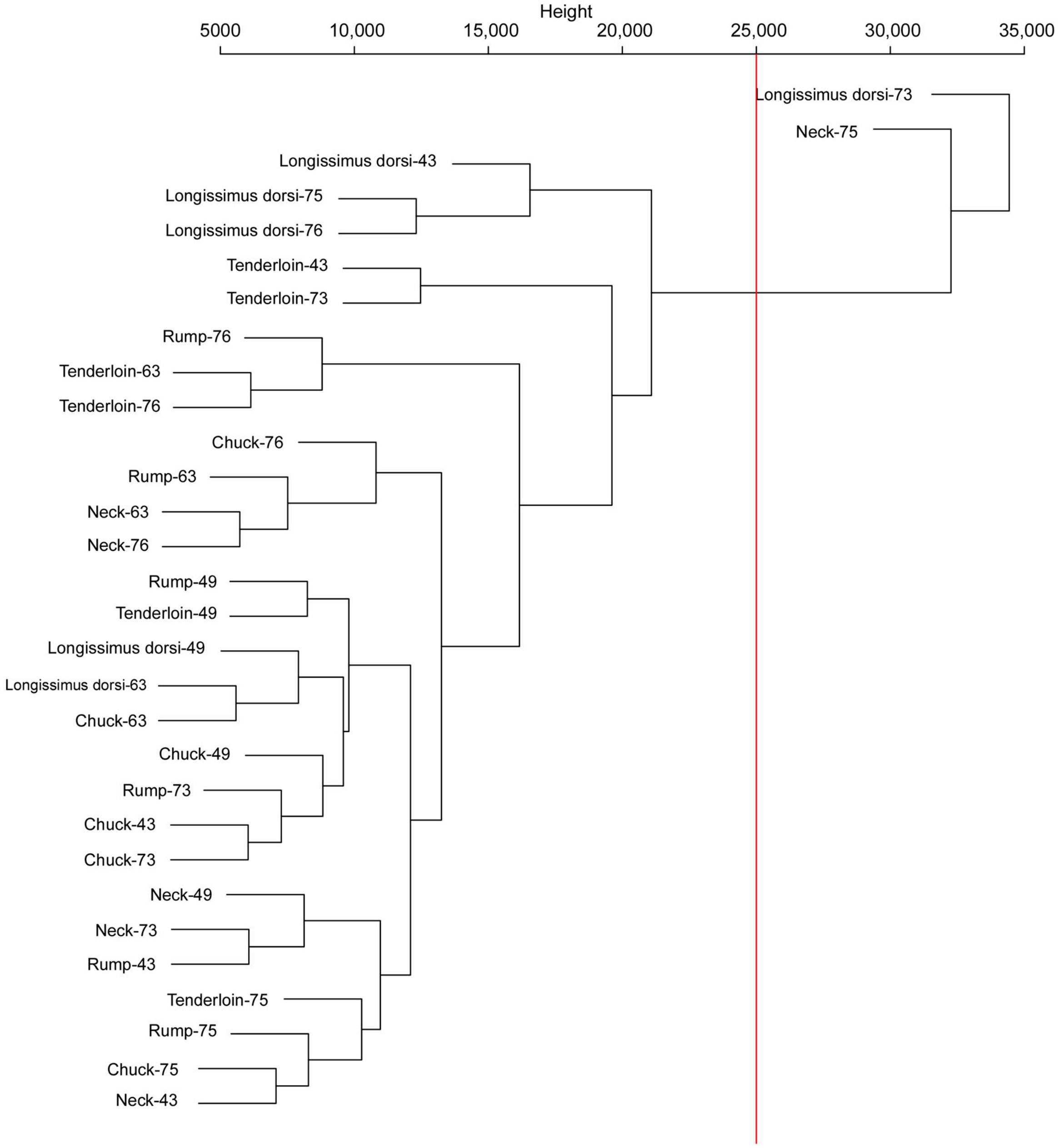

Figure S3: Hierarchical clustering of 30 samples based on the Euclidian distance using average linkage for agglomeration

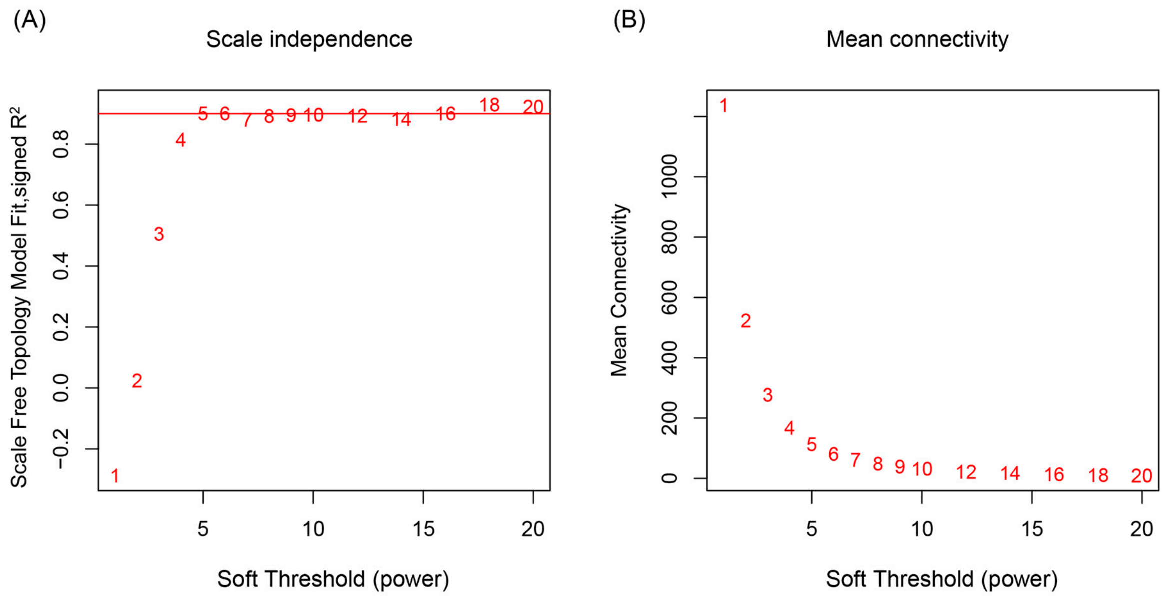

Figure S4: The determination of the power Beta (β) value is based on the scale free topology criterion (A) and mean connectivity (B) under the weighted gene correlation network analysis (WGCNA) method. The red line is R^2 equals 0.9.

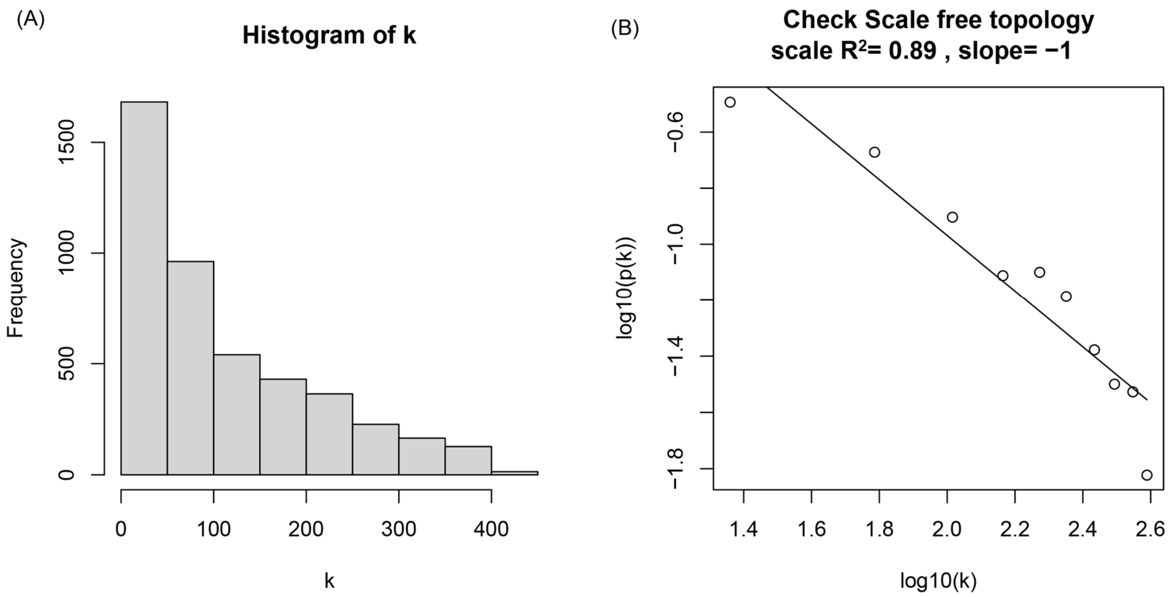

Figure S5: Network scale-free topology distribution test based on selected β value. (A) Gene connectivity frequency distribution map. K refers to the connectivity of each node in the network. (B) Check scale free topology. The round shape represents the relationship between log10 (k) and log10 (probability) of the frequency.

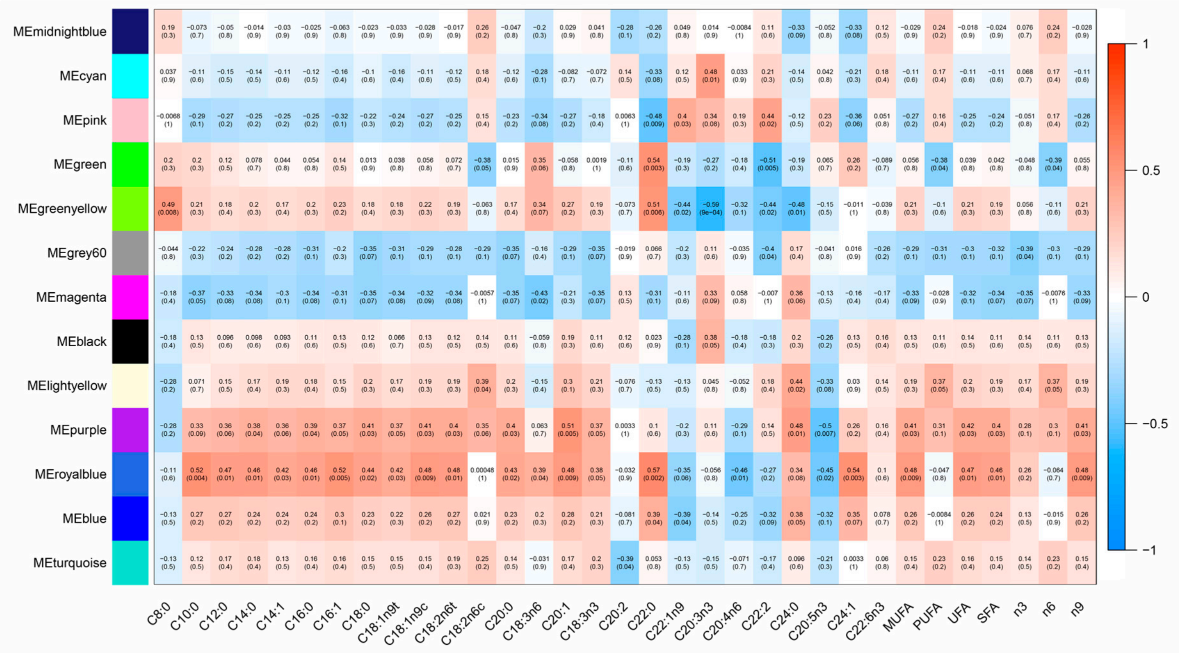

Figure S6: The heatmap of 13 modules and 33 fatty acid traits. Boxes contain Pearson correlation coefficients and their associated p values. Red color indicates that the given organization has a strong positive correlation to all other organizations. Blue color indicates that the given organization has a strong negative correlation to all other organizations.

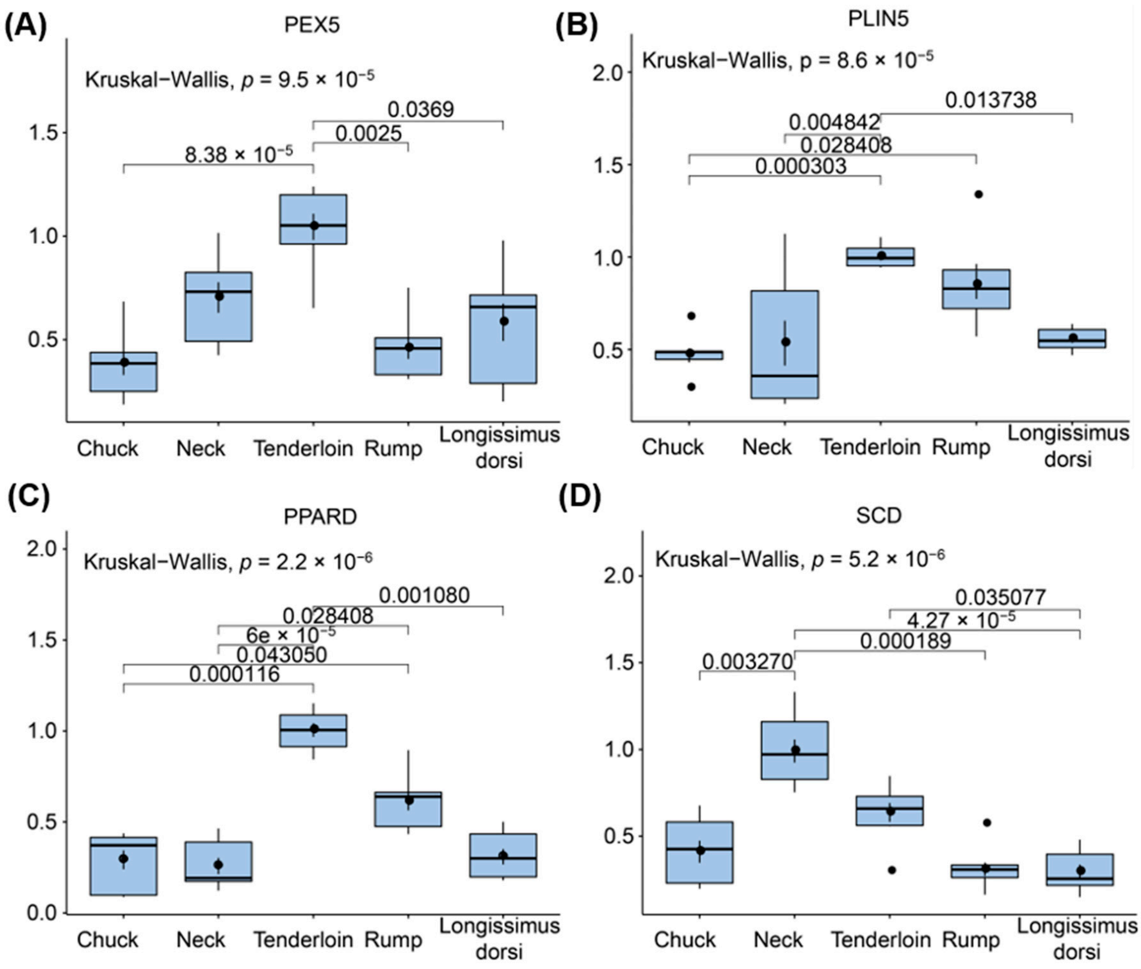

Figure S7: Validation of the expression levels of candidate genes related to fatty acid traits using RT-qPCR Boxplots show the mRNA expression levels of six candidate genes randomly selected in five types of beef cuts in Chinese Simmental beef cattle (n = 6). (A) *PEX5*, (B) *PLIN5*, (C) *PPARD*, (D) *SCD*. The ordinate is the relative expression level value, and the abscissa is the name of the bovine tissue sample. In the box plot: the maximum value (top of the line), the minimum value (low end of the line), the median (black point), the upper quartile (the upper border of the rectangle), the lower quartile (the bottom border of the rectangle), and the invalid data (out-of-line outliers).
