# Supplementary material for: Comparative Transcriptomic Analysis Reveals Diverse Expression Pattern Underlying Fatty Acid Composition among Different Beef Cuts
Source: Foods. 2022 Jan 4;11(1):117. doi: 10.3390/foods11010117 (PMC8750426; doi:10.3390/foods11010117)
Supplement: Supplementary file 1 [file foods-11-00117-s001.zip › Supplementary Table.pdf]

Supplementary Table S1. RT-qPCR primer sequences of candidate genes related to fatty acid traits in the beef cattle.

Gene name	Primer sequences (5'-3')		Sizes (bp)
PLIN5	F: GAGCTGGACTTTTGACCCGA	R: GTCCCCAACTCCACGCAATA	141
PEX5	F: GGGACTTGGAAGCCGAGAG	R: CCCCAAAGGCTTAGAGACCG	196
LPL	F: ACACAGCTGAGGACACTTGCC	R: GCCATGGATCACCACAAAGG	101
SCD	F: TCCTGTTGTTGTGCTTCATCC	R: GGCATAACGGAATAAGGTGGC	101
FABP3	F: GAACTCGACTCCCAGCTTGAA	R: AAGCCTACCACAATCATCGAAG	102
PPARD	F: ATGCACATGGTACTCACGCA	R: AGTTCCCGTCAGCCTCTTTG	77
ACSL3	F: CATCGTTTCTCTGGTCCCTC	R: TGCTAAGGGGTTTGTTTTCC	155
ELOVL5	F: AGAAGTACATCACGCAGGGC	R: ACCAACCGAGAGGGAAGGTA	99
FASN	F: ACCTCGTGAAGGCTGTGACTCA	R: TGAGTCGAGGCCAAGGTCTGAA	92
HSD17B8	F: GAACTTGGACGACACGGGAT	R: GCCAAGAATGCGACGACATC	167
GAPDH	F: CCTGCCCGTTCGACAGATA	R: GGCGACGATGTCCACTTTG	150

Supplement Table S2. Relative content of fatty acid components in chuck, neck, rump, tenderloin and longissimus dorsi.

Fatty acid composition	Chuck	Neck	Rump	Tenderloin	Longissimus dorsi
	Mean ± SD (g/100g)				
Saturated					
C8:0	0.006 ± 0.002	0.006 ± 0.002	0.006 ± 0.002	0.006 ± 0.002	0.002 ± 0.009
C10:0	0.001 ± 0.001	0.003 ± 0.003	0.001 ± 0.001	0.009 ± 0.004	0.004 ± 0.005
C12:0	0.002 ± 0.003	0.006 ± 0.005	0.002 ± 0.003	0.022 ± 0.011	0.011 ± 0.008
C14:0	0.129 ± 0.087	0.287 ± 0.179	0.101 ± 0.101	0.943 ± 0.493	0.493 ± 0.38
C16:0	2.858 ± 1.061	5.758 ± 3.04	2.584 ± 1.855	15.324 ± 6.945	6.945 ± 6.906
C18:0	1.576 ± 0.463	2.84 ± 1.296	1.371 ± 0.6	8.399 ± 3.696	3.696 ± 3
C20:0	0.009 ± 0.003	0.017 ± 0.008	0.008 ± 0.004	0.053 ± 0.026	0.026 ± 0.019
C22:0	0.011 ± 0.001	0.014 ± 0.001	0.012 ± 0.001	0.015 ± 0.002	0.002 ± 0.018

C24:0	0.009 ± 0.001	0.011 ± 0	0.01 ± 0.001	0.01 ± 0	0 ± 0.009
Monounsaturated					
C14:1	0.005 ± 0.003	0.011 ± 0.008	0.005 ± 0.005	0.027 ± 0.015	0.015 ± 0.014
C16:1	0.026 ± 0.01	0.061 ± 0.034	0.026 ± 0.02	0.116 ± 0.045	0.045 ± 0.071
C18:1n9t	0.028 ± 0.008	0.068 ± 0.038	0.028 ± 0.02	0.227 ± 0.128	0.128 ± 0.075
C18:1n9c	2.731 ± 0.734	6.147 ± 3.212	2.614 ± 1.752	12.892 ± 5.028	5.028 ± 6.274
C20:1	0.005 ± 0.001	0.014 ± 0.008	0.005 ± 0.003	0.022 ± 0.006	0.006 ± 0.009
C22:1n9	0.049 ± 0.005	0.035 ± 0.004	0.044 ± 0.007	0.041 ± 0.005	0.005 ± 0.035
C24:1	0.003 ± 0	0.005 ± 0.001	0.004 ± 0	0.005 ± 0	0 ± 0.005
Polyunsaturated					
C18:2n6t	0.003 ± 0.001	0.007 ± 0.004	0.003 ± 0.002	0.017 ± 0.006	0.006 ± 0.008
C18:2n6c	0.597 ± 0.046	0.576 ± 0.069	0.611 ± 0.051	0.789 ± 0.085	0.085 ± 0.386
C18:3n6	0.001 ± 0	0.002 ± 0	0.001 ± 0	0.003 ± 0.001	0.001 ± 0.003
C18:3n3	0.023 ± 0.002	0.025 ± 0.005	0.023 ± 0.004	0.045 ± 0.01	0.01 ± 0.026
C20:2	0.005 ± 0	0.005 ± 0.002	0.005 ± 0	0.004 ± 0.002	0.002 ± 0.005
C20:3n3	0.004 ± 0	0.005 ± 0.001	0.003 ± 0.001	0.003 ± 0	0 ± 0.003
C20:4n6	0.242 ± 0.015	0.193 ± 0.016	0.243 ± 0.023	0.205 ± 0.027	0.027 ± 0.19
C22:2	0.003 ± 0	0.002 ± 0	0.002 ± 0	0.003 ± 0.001	0.001 ± 0.001
C20:5n3	0.012 ± 0.001	0.008 ± 0.001	0.011 ± 0.002	0.01 ± 0.001	0.001 ± 0.011
C22:6n3	0.004 ± 0.001	0.003 ± 0.001	0.004 ± 0.001	0.009 ± 0.012	0.012 ± 0.008
Fatty acid groups					
MUFA	2.848 ± 0.756	6.341 ± 3.296	2.726 ± 1.796	13.328 ± 5.211	5.211 ± 6.482
PUFA	0.895 ± 0.046	0.825 ± 0.085	0.907 ± 0.055	1.087 ± 0.078	0.078 ± 0.641
UFA	3.742 ± 0.788	7.166 ± 3.338	3.633 ± 1.815	14.415 ± 5.282	5.282 ± 7.123
SFA	4.6 ± 1.605	8.942 ± 4.509	4.094 ± 2.56	24.78 ± 11.057	11.057 ± 10.354
n3	0.043 ± 0.003	0.041 ± 0.005	0.042 ± 0.004	0.066 ± 0.016	0.016 ± 0.047
n6	0.844 ± 0.044	0.777 ± 0.08	0.859 ± 0.052	1.014 ± 0.068	0.068 ± 0.587

n9	2.809 ± 0.743	6.25 ± 3.247	2.686 ± 1.768	13.16 ± 5.147	5.147 ± 6.384

Supplementary Table S3. Percentage of fatty acid components in chuck, neck, rump, tenderloin and longissimus dorsi.

Fatty acid composition	Chuck	Neck	Rump	Tenderloin	Longissimus dorsi
	Mean±SD (%)				
Saturated					
C8:0	0.078 ± 0.033	0.046 ± 0.035	0.078 ± 0.037	0.018 ± 0.012	0.055 ± 0.039
C10:0	0.012 ± 0.013	0.016 ± 0.013	0.006 ± 0.01	0.023 ± 0.003	0.033 ± 0.008
C12:0	0.019 ± 0.029	0.028 ± 0.022	0.013 ± 0.02	0.052 ± 0.01	0.05 ± 0.013
C14:0	1.354 ± 0.602	1.584 ± 0.502	1.028 ± 0.534	2.294 ± 0.391	2.053 ± 0.351
C16:0	32.147 ± 3.551	33.766 ± 3.718	30.015 ± 4.789	38.144 ± 1.797	37.996 ± 2.872
C18:0	18.158 ± 1.287	17.563 ± 1.095	17.727 ± 1.725	21.013 ± 2.147	16.769 ± 0.872
C20:0	0.103 ± 0.013	0.103 ± 0.012	0.107 ± 0.012	0.131 ± 0.014	0.107 ± 0.008
C22:0	0.131 ± 0.038	0.108 ± 0.062	0.176 ± 0.066	0.042 ± 0.011	0.117 ± 0.063
C24:0	0.109 ± 0.038	0.087 ± 0.054	0.145 ± 0.057	0.028 ± 0.012	0.058 ± 0.025
Monounsaturated					
C14:1	0.05 ± 0.019	0.061 ± 0.025	0.05 ± 0.027	0.066 ± 0.017	0.078 ± 0.024
C16:1	0.295 ± 0.038	0.353 ± 0.057	0.301 ± 0.06	0.296 ± 0.025	0.397 ± 0.044
C18:1n9t	0.326 ± 0.009	0.397 ± 0.045	0.327 ± 0.053	0.551 ± 0.102	0.404 ± 0.056
C18:1n9c	31.524 ± 2.08	36.567 ± 2.127	31.042 ± 3.494	32.827 ± 1.769	34.834 ± 1.87
C20:1	0.056 ± 0.006	0.083 ± 0.012	0.064 ± 0.004	0.057 ± 0.011	0.048 ± 0.027
C22:1n9	0.605 ± 0.196	0.29 ± 0.197	0.676 ± 0.283	0.121 ± 0.053	0.239 ± 0.127
C24:1	0.043 ± 0.011	0.035 ± 0.018	0.053 ± 0.018	0.014 ± 0.005	0.03 ± 0.013
Polyunsaturated					
C18:2n6t	0.038 ± 0.002	0.044 ± 0.007	0.04 ± 0.005	0.044 ± 0.004	0.042 ± 0.004

C18:2n6c	7.31 ± 2.024	4.461 ± 2.531	9.104 ± 3.471	2.233 ± 0.71	2.668 ± 2.331
C18:3n6	0.018 ± 0.005	0.012 ± 0.006	0.02 ± 0.008	0.008 ± 0.002	0.018 ± 0.004
C18:3n3	0.277 ± 0.062	0.18 ± 0.078	0.325 ± 0.103	0.12 ± 0.023	0.16 ± 0.044
C20:2	0.061 ± 0.02	0.038 ± 0.022	0.069 ± 0.025	0.012 ± 0.011	0.034 ± 0.021
C20:3n3	0.051 ± 0.014	0.037 ± 0.02	0.05 ± 0.019	0.008 ± 0.002	0.017 ± 0.016
C20:4n6	2.958 ± 0.735	1.525 ± 0.912	3.666 ± 1.43	0.625 ± 0.336	1.291 ± 0.666
C22:2	0.035 ± 0.015	0.018 ± 0.015	0.035 ± 0.017	0.007 ± 0.002	0.007 ± 0.007
C20:5n3	0.151 ± 0.042	0.066 ± 0.044	0.169 ± 0.067	0.029 ± 0.014	0.068 ± 0.033
C22:6n3	0.047 ± 0.015	0.023 ± 0.012	0.069 ± 0.036	0.024 ± 0.026	0.039 ± 0.032
Fatty acid groups					
MUFA	32.899 ± 2.058	37.785 ± 2.044	32.513 ± 3.333	33.931 ± 1.762	36.031 ± 1.798
PUFA	10.945 ± 2.873	6.402 ± 3.63	13.545 ± 5.119	3.109 ± 1.092	4.343 ± 2.772
UFA	43.844 ± 3.533	44.187 ± 2.175	46.058 ± 2.259	37.04 ± 2.547	40.374 ± 2.074
SFA	52.111 ± 4.378	53.302 ± 3.365	49.294 ± 3.785	61.747 ± 2.768	57.239 ± 2.872
n3	0.526 ± 0.124	0.305 ± 0.151	0.612 ± 0.215	0.181 ± 0.047	0.284 ± 0.071
n6	10.324 ± 2.719	6.041 ± 3.446	12.83 ± 4.868	2.909 ± 1.042	4.018 ± 2.699
n9	32.455 ± 2.043	37.253 ± 1.994	32.045 ± 3.269	33.499 ± 1.731	35.478 ± 1.819

Supplementary Table S4. Analysis of differential gene expression between chuck, neck, rump, tenderloin and longissimus dorsi tissues.

Group	Group of abbreviation	Up-regulation	Down-regulation	Number of DEGs
Longissimus dorsi vs chuck	ld vs ch	1791	2353	4144
Longissimus dorsi vs neck	ld vs ne	1608	2512	4120
Longissimus dorsi vs rump	ld vs ru	1601	2430	4031
Longissimus dorsi vs tenderloin	ld vs te	1587	2612	4199
Chuck vs neck	ch vs ne	422	826	1248
Chuck vs rump	ch vs ru	312	440	752
Chuck vs tenderloin	ch vs te	275	663	938
Neck vs rump	ne vs ru	477	351	828
Neck vs tenderloin	ne vs te	234	306	540
Rump vs tenderloin	ru vs te	152	278	430

Note: Number of differential genes identified based on reads count.

Supplementary Table S6. KEGG function enrichment analysis of differentially expressed genes among different groups.

Group		KEGG ID	Description	P value	Q value
Longissimus doris vs Chuck	Group1	bta03010	Ribosome	4.94×10^{15}	1.20×10^{12}
	Group1	bta00190	Oxidative phosphorylation	3.25×10^{13}	3.95×10^{11}
	Group1	bta04714	Thermogenesis	1.46×10^{12}	7.08×10^{11}
	Group1	bta04810	Regulation of actin cytoskeleton	9.94×10^{7}	1.87×10^{5}
	Group1	bta04020	Calcium signaling pathway	1.29×10^{3}	8.07×10^{3}
	Group1	bta01212	Fatty acid metabolism	2.13×10^{3}	1.23×10^{2}
	Group1	bta04260	Cardiac muscle contraction	1.26×10^{5}	1.61×10^{4}

Supplementary Table S5. GO function annotation analysis of differentially expressed genes among different groups.

Group		Category	GO ID	Description	P value	Q value
Longissimus doris vs Chuck	Group1	BP	GO:0006518	peptide metabolic process	4.67 × 10^{7}	7.31 × 10^{4}
	Group1	BP	GO:0006412	translation	5.87 × 10^{7}	7.31 × 10^{4}
	Group1	BP	GO:0043043	peptide biosynthetic process	9.64 × 10^{7}	7.31 × 10^{4}
	Group1	BP	GO:0043604	amide biosynthetic process	1.11 × 10^{6}	7.31 × 10^{4}
	Group1	BP	GO:0043603	cellular amide metabolic process	6.68 × 10^{6}	3.53 × 10^{3}
	Group1	CC	GO:0098798	mitochondrial protein complex	4.17 × 10^{16}	1.39 × 10^{13}
	Group1	CC	GO:0005840	ribosome	3.18 × 10^{11}	5.30 × 10^{9}
	Group1	CC	GO:0098800	inner mitochondrial membrane protein complex	5.01 × 10^{11}	5.55 × 10^{9}
	Group1	CC	GO:0098803	respiratory chain complex	2.58 × 10^{10}	1.87 × 10^{8}
	Group1	CC	GO:0005743	mitochondrial inner membrane	2.80 × 10^{10}	1.87 × 10^{8}
	Group1	MF	GO:0003735	structural constituent of ribosome	3.19 × 10^{7}	1.33 × 10^{4}
	Group1	MF	GO:0005198	structural molecule activity	3.65 × 10^{4}	4.67 × 10^{2}
	Group1	MF	GO:0003954	NADH dehydrogenase activity	5.71 × 10^{4}	4.67 × 10^{2}
	Group1	MF	GO:0016655	oxidoreductase activity, acting on NAD(P)H, quinone or similar compound as acceptor	5.71 × 10^{4}	4.67 × 10^{2}
	Group1	MF	GO:0008137	NADH dehydrogenase (ubiquinone) activity	6.71 × 10^{4}	4.67 × 10^{2}
Longissimus doris vs Neck	Group2	BP	GO:0033108	mitochondrial respiratory chain complex assembly	1.13 × 10^{9}	2.91 × 10^{6}
	Group2	BP	GO:0010257	NADH dehydrogenase complex assembly	3.33 × 10^{9}	2.91 × 10^{6}
	Group2	BP	GO:0006412	translation	3.32 × 10^{5}	1.45 × 10^{2}
	Group2	BP	GO:0006518	peptide metabolic process	5.88 × 10^{5}	2.00 × 10^{2}
	Group2	BP	GO:0043604	amide biosynthetic process	2.02 × 10^{4}	4.80 × 10^{2}
	Group2	CC	GO:0098798	mitochondrial protein complex	2.69 × 10^{28}	9.42 × 10^{26}

	Group2	CC	GO:0098803	respiratory chain complex	2.34×10^{14}	1.17×10^{12}
	Group2	CC	GO:1990204	oxidoreductase complex	2.10×10^{8}	3.06×10^{7}
	Group2	CC	GO:0030315	T-tubule	6.95×10^{5}	8.99×10^{4}
	Group2	CC	GO:0015935	small ribosomal subunit	2.13×10^{3}	2.25×10^{2}
	Group2	MF	GO:0003735	structural constituent of ribosome	7.07×10^{6}	2.38×10^{3}
	Group2	MF	GO:0003954	NADH dehydrogenase activity	1.15×10^{5}	2.38×10^{3}
	Group2	MF	GO:0008137	NADH dehydrogenase (ubiquinone) activity	8.87×10^{5}	9.17×10^{3}
	Group2	MF	GO:0050136	NADH dehydrogenase (quinone) activity	8.87×10^{5}	9.17×10^{3}
	Group2	MF	GO:0016655	oxidoreductase activity, acting on NAD(P)H, quinone or similar compound as acceptor	4.53×10^{4}	3.75×10^{2}
	Group3	BP	GO:0033108	mitochondrial respiratory chain complex assembly	2.00×10^{8}	5.24×10^{5}
	Group3	BP	GO:0006518	peptide metabolic process	9.52×10^{8}	6.67×10^{5}
	Group3	BP	GO:0006412	translation	3.33×10^{7}	1.75×10^{4}
	Group3	BP	GO:0043604	amide biosynthetic process	3.34×10^{6}	1.25×10^{3}
	Group3	BP	GO:0034622	cellular protein-containing complex assembly	1.36×10^{4}	3.23×10^{2}
Longissimus doris vs Rump	Group3	CC	GO:0098798	mitochondrial protein complex	1.31×10^{23}	4.42×10^{21}
	Group3	CC	GO:0098803	respiratory chain complex	3.06×10^{15}	2.07×10^{13}
	Group3	CC	GO:1990204	oxidoreductase complex	1.97×10^{10}	3.70×10^{9}
	Group3	CC	GO:0030315	T-tubule	4.75×10^{3}	4.22×10^{2}
	Group3	CC	GO:0045259	proton-transporting ATP synthase complex	4.45×10^{3}	4.06×10^{2}
	Group3	MF	GO:0003735	structural constituent of ribosome	1.21×10^{8}	5.07×10^{6}
	Group3	MF	GO:0005198	structural molecule activity	5.54×10^{5}	1.17×10^{2}

	Group3	MF	GO:0003954	NADH dehydrogenase activity	2.85×10^{4}	2.95×10^{2}
	Group3	MF	GO:0008137	NADH dehydrogenase (ubiquinone) activity	3.50×10^{4}	2.95×10^{2}
	Group3	MF	GO:0050136	NADH dehydrogenase (quinone) activity	3.50×10^{4}	2.95×10^{2}
	Group4	BP	GO:0033108	mitochondrial respiratory chain complex assembly	1.33×10^{7}	3.51×10^{4}
	Group4	BP	GO:0006518	peptide metabolic process	7.79×10^{6}	5.12×10^{3}
	Group4	BP	GO:0006412	translation	1.46×10^{5}	7.66×10^{3}
	Group4	BP	GO:0007519	skeletal muscle tissue development	1.15×10^{4}	3.03×10^{2}
	Group4	BP	GO:0060538	skeletal muscle organ development	1.15×10^{4}	3.03×10^{2}
	Group4	CC	GO:0098798	mitochondrial protein complex	1.42×10^{15}	4.74×10^{13}
Longissimus doris vs Tenderloin	Group4	CC	GO:0098803	respiratory chain complex	3.76×10^{10}	2.51×10^{8}
	Group4	CC	GO:1990204	oxidoreductase complex	4.62×10^{7}	8.11×10^{6}
	Group4	CC	GO:0030315	T-tubule	5.08×10^{5}	7.37×10^{4}
	Group4	CC	GO:0009986	cell surface	2.90×10^{3}	2.93×10^{2}
	Group4	MF	GO:0003735	structural constituent of ribosome	1.15×10^{4}	4.60×10^{2}
	Group4	MF	GO:0003954	NADH dehydrogenase activity	3.68×10^{4}	4.60×10^{2}
	Group4	MF	GO:0008137	NADH dehydrogenase (ubiquinone) activity	4.45×10^{4}	4.60×10^{2}
	Group4	MF	GO:0050136	NADH dehydrogenase (quinone) activity	4.45×10^{4}	4.60×10^{2}
	Group5	BP	GO:0009135	purine nucleoside diphosphate metabolic process	1.94×10^{5}	1.46×10^{2}
	Group5	BP	GO:0006091	generation of precursor metabolites and energy	3.45×10^{5}	1.75×10^{2}
Chuck vs Neck	Group5	BP	GO:0005975	carbohydrate metabolic process	3.90×10^{5}	1.75×10^{2}
	Group5	BP	GO:0007009	plasma membrane organization	9.25×10^{5}	2.97×10^{2}
	Group5	BP	GO:0019752	carboxylic acid metabolic process	1.61×10^{4}	3.70×10^{2}

Group5	CC	GO:0019866	organelle inner membrane	1.55×10^{4}	2.58×10^{2}
Group5	CC	GO:0005740	mitochondrial envelope	1.86×10^{4}	2.58×10^{2}
Group5	CC	GO:0031966	mitochondrial membrane	2.50×10^{4}	2.58×10^{2}
Group5	CC	GO:0005743	mitochondrial inner membrane	4.60×10^{4}	3.57×10^{2}

Longissimus doris vs Neck	Group1	bta04270	Vascular smooth muscle contraction	1.97×10^{4}	1.65×10^{3}
	Group1	bta04151	PI3K-Akt signaling pathway	1.19×10^{3}	7.64×10^{3}
	Group1	bta04310	Wnt signaling pathway	1.35×10^{3}	8.22×10^{3}
	Group2	bta00190	Oxidative phosphorylation	2.69×10^{18}	4.31×10^{16}
	Group2	bta04714	Thermogenesis	1.79×10^{16}	7.00×10^{15}
	Group2	bta04260	Cardiac muscle contraction	1.38×10^{6}	2.50×10^{5}
	Group2	bta04022	cGMP-PKG signaling pathway	4.35×10^{5}	5.68×10^{4}
	Group2	bta04670	Leukocyte transendothelial migration	6.96×10^{5}	8.16×10^{4}
	Group2	bta04810	Regulation of actin cytoskeleton	1.07×10^{4}	1.14×10^{3}
	Group2	bta04151	PI3K-Akt signaling pathway	4.31×10^{4}	4.40×10^{3}
	Group2	bta04020	Calcium signaling pathway	6.87×10^{4}	6.02×10^{3}
	Group2	bta04066	HIF-1 signaling pathway	2.52×10^{3}	1.62×10^{2}
	Group2	bta04270	Vascular smooth muscle contraction	4.67×10^{3}	2.61×10^{2}
Longissimus doris vs Rump	Group3	bta04714	Thermogenesis	1.63×10^{20}	3.78×10^{18}
	Group3	bta00190	Oxidative phosphorylation	7.34×10^{18}	8.50×10^{16}
	Group3	bta03010	Ribosome	5.96×10^{16}	4.60×10^{14}
	Group3	bta04810	Regulation of actin cytoskeleton	5.76×10^{7}	7.41×10^{6}
	Group3	bta04921	Oxytocin signaling pathway	1.81×10^{6}	2.00×10^{5}

	Group3	bta04270	Vascular smooth muscle contraction	3.00×10^{5}	2.78×10^{4}
	Group3	bta04151	PI3K-Akt signaling pathway	1.13×10^{3}	7.49×10^{3}
	Group3	bta01212	Fatty acid metabolism	4.35×10^{3}	2.05×10^{2}
	Group3	bta04020	Calcium signaling pathway	8.79×10^{3}	3.28×10^{2}
	Group3	bta04923	Regulation of lipolysis in adipocytes	9.92×10^{3}	3.53×10^{2}
	Group4	bta04714	Thermogenesis	6.88×10^{14}	1.64×10^{11}
	Group4	bta00190	Oxidative phosphorylation	2.11×10^{10}	1.01×10^{8}
	Group4	bta03010	Ribosome	2.66×10^{9}	7.06×10^{8}
	Group4	bta04810	Regulation of actin cytoskeleton	1.74×10^{7}	2.78×10^{6}
Longissimus doris vs Tenderloin	Group4	bta04270	Vascular smooth muscle contraction	4.79×10^{5}	4.98×10^{4}
	Group4	bta04260	Cardiac muscle contraction	1.70×10^{4}	1.51×10^{3}
	Group4	bta04151	PI3K-Akt signaling pathway	1.11×10^{3}	7.78×10^{3}
	Group4	bta04730	Long-term depression	1.14×10^{3}	7.78×10^{3}
	Group4	bta01212	Fatty acid metabolism	1.32×10^{3}	8.77×10^{3}
	Group4	bta04724	Glutamatergic synapse	7.56×10^{3}	3.39×10^{2}
	Group5	bta01200	Carbon metabolism	5.40×10^{7}	1.45×10^{4}
	Group5	bta01230	Biosynthesis of amino acids	1.11×10^{6}	1.49×10^{4}
	Group5	bta00010	Glycolysis / Gluconeogenesis	1.25×10^{4}	7.07×10^{3}
Chuck vs Neck	Group5	bta00020	Citrate cycle (TCA cycle)	1.32×10^{4}	7.07×10^{3}
	Group5	bta00500	Starch and sucrose metabolism	2.15×10^{4}	8.25×10^{3}
	Group5	bta00340	Histidine metabolism	8.20×10^{4}	2.24×10^{2}
	Group5	bta04020	Calcium signaling pathway	9.80×10^{4}	2.24×10^{2}

Group5	bta04714	Thermogenesis	1.00×10^{3}	2.24×10^{2}
Group5	bta04925	Aldosterone synthesis and secretion	1.74×10^{3}	3.34×10^{2}
Group5	bta00260	Glycine,serine and threonine metabolism	2.39×10^{3}	4.28×10^{2}

Supplementary Table S7. The identified functional modules using dynamic cutting method.

Module	Number of module genes
Black	366
Blue	549
Cyan	191
Green	484
Greenyellow	151
Grey60	152
Lightyellow	59
Magenta	163
Midnightblue	93
Pink	255
Purple	157

Royalblue	447
Turquoise	1443

Supplementary Table S8. Functional annotation enrichment analysis of fatty acid-related genes.

Category	Term ID	Term description	False discovery rate
	GO:0006631	fatty acid metabolic process	3.41×10^{20}
	GO:0016042	lipid catabolic process	4.11×10^{8}
	GO:0006633	fatty acid biosynthetic process	4.61×10^{8}
	GO:0006635	fatty acid beta-oxidation	5.90×10^{8}
BP	GO:0044539	long-chain fatty acid import into cell	1.56×10^{5}
	GO:0019216	regulation of lipid metabolic process	2.27×10^{5}
	GO:0010884	positive regulation of lipid storage	5.19×10^{5}
	GO:0044249	cellular biosynthetic process	6.69×10^{5}
	GO:1901575	organic substance catabolic process	1.10×10^{4}
	GO:0046889	positive regulation of lipid biosynthetic process	7.40×10^{4}
	GO:0005737	cytoplasm	1.02×10^{6}
	GO:0005739	mitochondrion	5.43×10^{6}
CC	GO:0043227	membrane-bounded organelle	5.43×10^{6}
	GO:0043231	intracellular membrane-bounded organelle	5.43×10^{6}
	GO:0005623	cell	9.39×10^{6}
	GO:0016627	oxidoreductase activity, acting on the CH-CH group of donors	8.40×10^{7}
	GO:0016746	transferase activity, transferring acyl groups	1.17×10^{6}
	GO:0003824	catalytic activity	1.27×10^{6}
MF	GO:0016628	oxidoreductase activity, acting on the CH-CH group of donors, NAD or NADP as acceptor	1.27×10^{6}
	GO:0016747	transferase activity, transferring acyl groups other than amino-acyl groups	6.95×10^{6}
	GO:0016616	oxidoreductase activity, acting on the CH-OH group of donors, NAD or NADP as acceptor	1.70×10^{4}
	GO:0004095	carnitine O-palmitoyltransferase activity	4.40×10^{4}

	GO:0004312	fatty acid synthase activity	8.00×10^{4}
	GO:0016491	oxidoreductase activity	8.00×10^{4}
	GO:0016740	transferase activity	2.20×10^{3}
KEGG	bta01212	Fatty acid metabolism	4.97×10^{20}
	bta03320	PPAR signaling pathway	1.57×10^{18}
	bta00071	Fatty acid degradation	1.05×10^{12}
	bta00061	Fatty acid biosynthesis	4.78×10^{7}
	bta01100	Metabolic pathways	5.41×10^{7}
	bta04146	Peroxisome	9.42×10^{6}
	bta04152	AMPK signaling pathway	5.36×10^{5}
	bta04920	Adipocytokine signaling pathway	1.50×10^{4}
	bta01040	Biosynthesis of unsaturated fatty acids	1.90×10^{4}
	bta00062	Fatty acid elongation	2.40×10^{4}
